# Late antenatal care utilization in Ethiopia: The effect of socio-economic inequities and regional disparities

**DOI:** 10.1371/journal.pgph.0000584

**Published:** 2022-11-29

**Authors:** Belayneh Mengist, Bekalu Endalew, Gedefaw Diress, Amanuel Abajobir

**Affiliations:** 1 Department of Public Health, College of Health Sciences, Debre Markos University, Debre Markos, Ethiopia; 2 African Population and Health Research Center, Nairobi, Kenya; PLOS: Public Library of Science, UNITED STATES

## Abstract

Antenatal care (ANC) is one of the most crucial components of maternal health care services. However, less than two-third of pregnant women receive ANC at least once and only 32% had at least 4 ANC visits in Ethiopia. There is dearth of nationally representative data that indicate changes in utilization of ANC services at the end of health sector transformation plan I period (HSTP I) in the country. Therefore, the present study aimed to investigate utilization of ANC the effect of socio-economic inequities and regional disparities in Ethiopia. The 2019 Ethiopian Mini Demographic and Health Survey data were used. A total of 5753 women in the reproductive age who gave live births in the five years preceding the survey were used for this study. Multivariable logistic regression model was fitted to identify factors associated with ANC booking. This study indicated that 74% women had at least one ANC visit during their last pregnancy of which four out of ten did not receive the recommended 4+ visits. The proportion of women who had late ANC booking (i.e., first ANC visit to health facility after 4 months of pregnancy) was found to be 32% and significant disparities were observed across regions. Rural residency (adjusted OR (AOR): 1.62, 95% CI (1.28, 2.05)), being wealth (AOR: 0.69, 95% CI (0.55, 0.85)), education (AOR: 0.25, 95% CI (0.15, 0.40)) and being grand multi-parity (AOR: 1.35, 95% CI (1.005, 1.83)) were significantly associated with late ANC booking. ANC services utilization is far behind its targets and the proportion of pregnant women entering ANC late is high. It is necessary to intensify efforts to raise awareness about the need of early ANC initiation, particularly in rural areas.

## Introduction

According to World Health Organization (WHO), antenatal care (ANC) is care provided by skilled health care providers to expectant woman from the beginning of pregnancy until the onset of labor. It is a key entry point for pregnant women in order to prepare for birth and parenthood as well as to prevent, detect, alleviate, or manage health problems during pregnancy that affect pregnant women and babies. These are complications of pregnancy itself, pre-existing conditions that worsen during pregnancy and effects of unhealthy lifestyles [1]. Pregnant women attending ANC visits receive sufficient evidence-based clinical interventions, such as deworming, iron and folic acid supplements, tetanus toxoid vaccination, counseling on maternal health, emergency preparedness, management of sexually transmitted infections, administration of antiretroviral therapy in HIV-positive women, supply of essential information about improved hygiene practices and the risks associated with pregnancy and childbirth [2].

In 2017, 295, 000 women died during and after pregnancy and childbirth, which is an unacceptable level of maternal mortality. Of these deaths, 94% occurred in low-resource settings, and most could have been prevented. Sub-Saharan Africa accounted for roughly two-thirds (196, 000) of maternal deaths [3]. According to 2016 Ethiopian Demography and Health Survey (EDHS), the maternal mortality ratio (MMR) for Ethiopia was 412 deaths per 100,000 live births, which is nearly the same with MEDHS 2014 [4]. The main causes of maternal mortality in Ethiopia were postpartum haemorrhage, sepsis, pre-eclampsia and eclampsia and birth complications [5]. Although most maternal deaths are preventable, many women often do not have access to evidence-based interventions such as the use of ANC services during pregnancy, and related services in childbirth and the postpartum period due to poverty, lack of information, and cultural barriers [6].

Evidence from the 2016 EDHS indicates that 62% of pregnant women received ANC of which only 32% received four or more ANC visits, which is very low as compared to the standard stetted. The recently developed second Health Sector Transformation Plan (HSTP II) that is being implemented from 2020 to 2025 sought agenda to reduce maternal mortality to 70 deaths per 100,000 live births, and one of the strategies to achieve the goal is the improvement in ANC services utilization among pregnant women [7]. While Ethiopia has made efforts to reduce maternal mortality through improved ANC use [8–10], it remains uncertain whether appropriate ANC use improved substantially in the context of the high maternal mortality in the country and the renewed global commitment to reduce maternal mortality to less than 70 per 100,000 births by 2030 Sustainable Development Goal (SDG) III.

Moreover, the assessment of factors associated with any change in the utilization of ANC services use during the end of HSTP I period would be helpful to Ethiopian public health experts and policy-makers to refine current maternal health interventions for high-risk populations. Studies were conducted on ANC in Ethiopia. However, those studies do not provide up-to-date evidence as they were conducted before the implementations of HSTP I (2015/16). The HSTP I was planned by the Federal Democratic Republic of Ethiopia Ministry of Health with an ambitious aim of reducing maternal mortality to 267 per 100,000 live births, a set of high impact interventions were being implemented, including antenatal care (ANC), skilled birth services and postnatal (PNC). It was planned to increase the proportion of pregnant women having at least 4 visits of ANC to 95% [11].

To the best of authors’ knowledge, there is no recent nationally representative study that assesses utilization of ANC services during the end of HSTP I to inform current priorities in Ethiopia. In addition regional disparity in terms of utilization as well as initiation of ANC in Ethiopia is not yet investigated. Therefore, the present study aimed to investigate the utilization of ANC, the effect of socio-economic inequities and regional disparities in Ethiopia using the 2019 mini EDHS (EMDHS).

## Methods

This is an observational (survey) study conducted in Ethiopia. Ethiopia share borders with Eritrea, Djibouti, Somalia, Sudan, South Sudan, and Kenya in the horn of Africa. The country covers an area of 1.1 million square kilometers and divided into twelve regional states and two city administrations further subdivided into 68 zones, 817 districts, and 16,253 kebeles (the lowest administrative units). According to the 2018 World Bank report, Ethiopia had a total population of 109 million [12].

### Source of data

The 2019 EMDHS (the second mini EDHS) data were collected from 21 March to 28 June 2019. Datasets were downloaded after the approval of Measure DHS (http://www.measuredhs.com), and specifically women’s file (IR) was used. The 2019 EMDHS sample was stratified and selected in two stages, and interviews were conducted face-to-face with permanent residents and visitors who stayed in the residences the day before the survey. The 2019 EMDHS sampling frame was a composite of all census enumeration areas (EAs) (based on the 2019 Ethiopia Population and Housing Census (PHC) frame. An EA is a geographical area with an average of 131 households. The sampling frame includes data on the EA’s location, type of residence (urban or rural), and the estimated number of residential households [13].

In the first stage, a total of 305 EAs (93 in urban areas and 212 in rural areas) were selected with probability proportional to EA size) and with independent selection in each sampling stratum. In the second stage, a fixed number of 30 households per cluster were selected with an equal probability systematic selection. All women age 15–49, who were either permanent residents of the selected households or visitors who slept in the household the night before the survey, were eligible to be interviewed. In all selected households, women aged 15–49 years were interviewed using the Woman’s Questionnaire.

The 2019 EMDHS data were pre-tested before the actual data collection. Data collectors had received training in interviewing techniques, field procedures, the content of the questionnaires, and how to administer both paper and electronic questionnaires; after all, questionnaires were finalized in English, and then translated into Amarigna, Tigrigna, and Oromiffa [13]. The sample consisted of 5753 women of age 15–49 years who had live births in the five years preceding the survey. The analytic sample for the current study consisted of 2908 women who had at least one ANC visit to a health facility in the last five years prior to the survey.

### Study variables

A binary response outcome variable was the first ANC booking/initiation to the health facility within five years of the survey which was coded as “0” if early and “1” if late. ANC booking was taken as early if initiated before 4 months and late if then after [14]. The quality of ANC services utilization was assessed using the five essential components of ANC services that every pregnant woman should get. The five components considered were measuring blood pressure, taking sample of blood, taking sample of urine, giving iron tablets/syrup, and informing about pregnancy complications (counseling about danger signs of pregnancy) [15].

The explanatory variables were women’s age and education level, place of residence, household wealth index (poor, middle and rich), parity and marital status. Households wealth index were derived using principal component analysis based on the number and kinds of consumer goods they own [13].

### Data management and statistical analysis

Since this was secondary data, the data were maintained by processing, editing, coding, and re-coding, checking completeness, and cleaning the missing values by running frequencies based on research question(s) using STATA version 14 statistical software. Cross tabulations and summary statistics were used to describe the study population. Logistic regression model was fitted to identify factors associated with ANC booking during pregnancy. Bivariate logistic regression was carried out and variables with a p-value <0.25 were selected multi variable analysis. The final multivariable logistic regression model fitness was checked using Hosmer and Lemeshow goodness of fit test statistics. A p-value < 0.05 was taken to declare statistically significant factors and odds ratio with 95% confidence interval was used to assess the strength of association.

### Ethics statement

An authorization letter to access the EDHS-2019 data set was obtained from the MEASURE DHS database at (http://www.measuredhs.com). We obeyed the terms and conditions of data sharing policy; data were used for the current study only, and kept confidential. Complete information regarding the ethical issue is available in the EDHS-2019 report [13].

## Results

### Socio-demographic characteristics

The mean age of study participants (n = 3952) was 28.6 year, with just 6% those were aged 15 to 19 years old and 75% of women were from rural residence. Three thousand six hundred fifty nine (93%) of the participants were married; whereas 29(0.73%) were never married. Nearly 52% of women had no formal education and nearly half (47%) were reported to have poor wealth index (**Table 1).**

**Table 1:** Socio-demographic characteristics of women in Ethiopia (n = 3952).

Variable	Frequency	Percent
**Women’s age (in years)**		
15–19	246	6.22
20–29	2019	51.09
30–39	1354	34.26
40–49	333	8.43
**Place of residence**		
Urban	999	25.28
Rural	2953	74.72
**Marriage**		
Never married	29	0.73
Married	3659	92.59
Widowed	44	1.11
Divorced	220	5.57
**Women’s educational Level**		
No education	2046	51.77
Primary	1300	32.89
Secondary	374	9.46
Higher	232	5.87
**Household wealth index**		
Poor	1858	47.01
Middle	583	14.75
Rich	1511	38.23
**Religion**		
Orthodox	1253	31.71
Catholic	25	0.63
Protestant	775	19.61
Muslim	1851	46.84
Traditional	35	0.89
Other	13	0.33

### Utilization and quality of antenatal care

Eight hundred forty seven (21.5%) study participants were premi-parous women. From the total study participants, 26% (95%CI: 25.1, 27.8) of pregnant women did not get ANC services and the proportion of women who had at least four ANC visits during their last pregnancy was 57%. The proportion of women who had early ANC booking (before 4 months) during their last pregnancy was found to be 68% (95%CI: 66.3, 69.7).

The quality of ANC services utilization was assessed using the five essential components of ANC services (indicators). From women who had ANC services, blood pressure measurement, urine and blood tests were done for 90%, 79% and 83%, respectively. About 58% of women received iron tablets/syrup of which only 19% of pregnant women took for three or more months. Forty three percent of the pregnant women did not get counseling about danger signs of pregnancy (**Table 2)**.

**Table 2:** Antenatal care service utilization and obstetric characteristics of women in Ethiopia (n = 3952).

Variables	Frequency	Percent
**Parity (children ever born)**		
1	847	21.49
2–4	1777	44.96
5 or more	1328	33.60
**Number of ANC visit(s)**		
No visit	1044	26.42
One-three visit	1244	31.48
Four and greater	1650	41.75
Don’t know	14	0.35
**Counseling given by health worker**		
No	820	28.20
Yes	2088	71.80
**ANC booking**		
Early	1979	68.1
Lately	929	31.95
**Blood pressure taken**		
No	277	9.53
Yes	2631	90.47
**Urine sample taken**		
No	615	21.15
Yes	2293	78.85
**Blood sample taken**		
No	496	17.06
Yes	2412	82.94
**Given iron tablets/syrup**		
No	1651	41.78
Yes	2298	57.92
Don’t know	12	0.30
**Days tablets or syrup taken (in days**)		
Not taken	44	1.92
<30	478	20.88
30–59	828	36.17
60–89	475	20.75
≥90	436	19.05
Don’t know	28	1.22
**Told about signs of pregnancy**		
No	1251	43.02
Yes	1646	56.60
Don’t know	11	0.38

One third of pregnant women in Ethiopia did not get counseling about vaginal bleeding during pregnancy, and only 29% and 36% of pregnant women received counseling about blurred vision and abdominal pain respectively (**Table 3**).

**Table 3:** Counseling on danger signs of pregnancy among women who received antenatal care service in Ethiopia.

Danger signs	No	Yes
	Frequency (%)	Frequency (%)
Vaginal bleeding	548 (33.29)	1098 (66.71)
Vaginal gush of fluid	1111(67.50)	535 (32.50)
Severe headache	794 (48.24)	852 (51.76)
Blurred vision	1164 (70.72)	482 (29.28)
Fever	1254 (76.18)	392 (23.82)
Abdominal pain	1054 (64.03)	592 (35.97)
Convulsion	1456 (88.46)	190 (11.54)
Other	1556 (94.65)	88 (5.35)

## Regional disparities of ANC services utilization in Ethiopia

**Fig 1:**
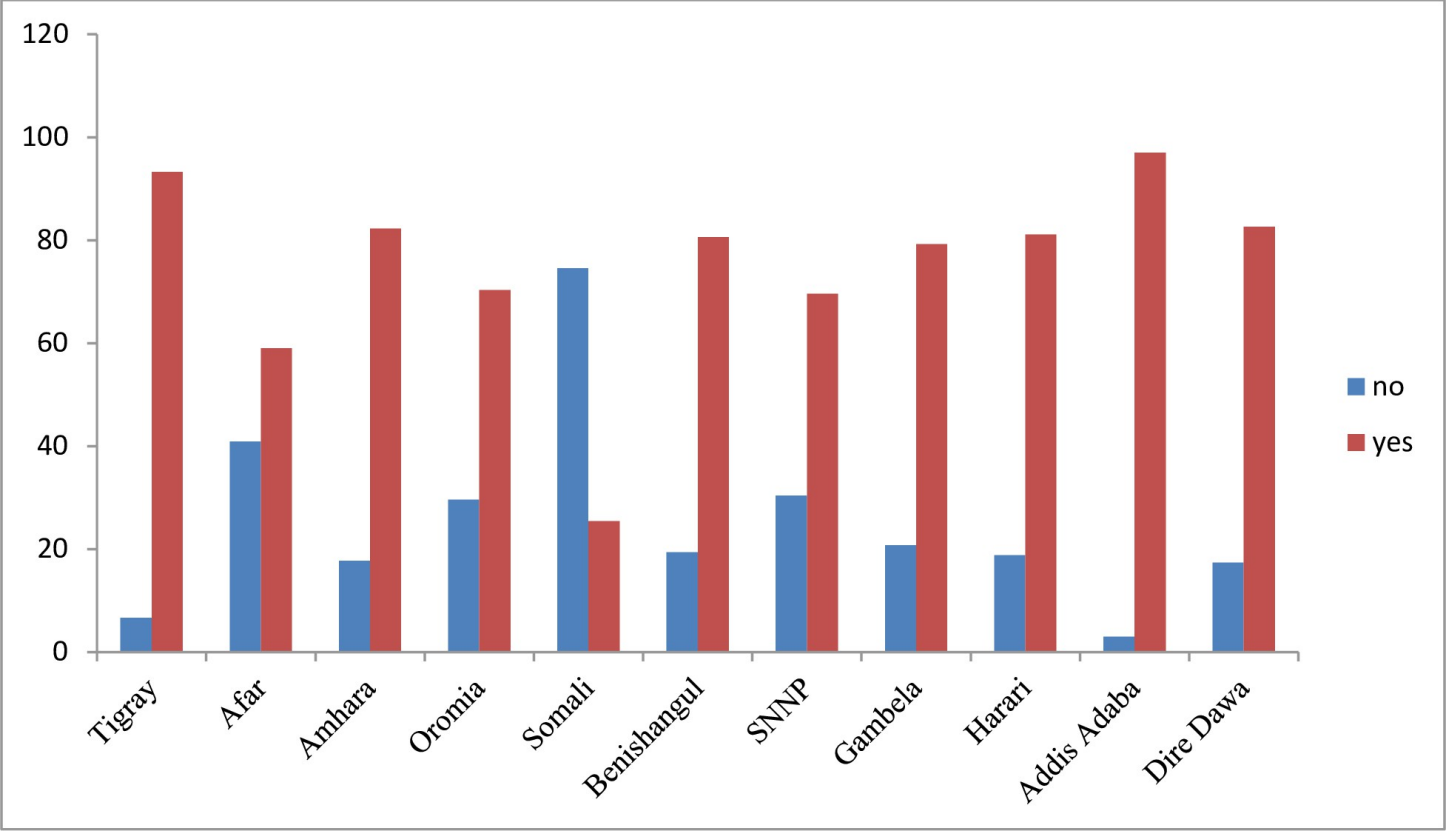
Regional disparities of ANC services utilization among women in Ethiopia, 2019.

Antenatal care services utilization varied across regions in Ethiopia. The proportion of ANC service utilization was as low as 25% in Somali region and as high as 96% in Addis Ababa (**Fig 1).** In addition, the proportion of pregnant women who had four or more ANC visits was highest in Addis Ababa (82%) followed by Tigray (60%) and Dire Dawa (59%) and was lowest in Somali (9%) followed by Gambela (28%) region.

**Fig 2:**
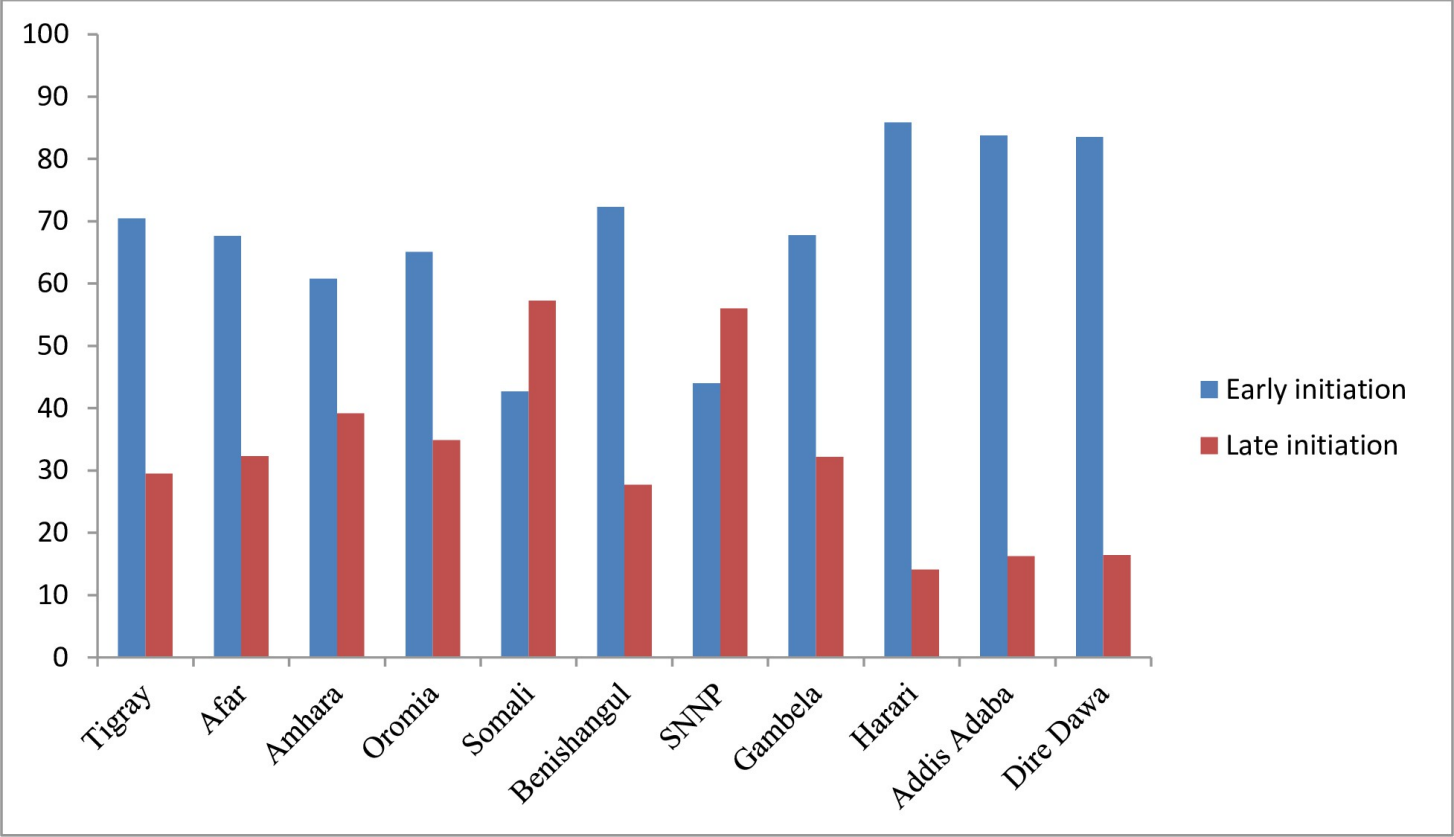
Time of ANC booking among women by regions in Ethiopia, 2019.

Regional disparities were also observed on time of ANC initiation/booking. Early ANC booking was observed in Harari region (86%), Addis Ababa (84%) and Dire Dawa (83.5%) town administrations whereas late ANC booking was highly observed in Somali (42.7%) followed by South Nation Nationalities and Peoples (SNNP) (44%) region **(Fig 2).**

### Factors associated with ANC booking in Ethiopia

In multivariable analyses of factors associated with late ANC booking among pregnant women, six explanatory variables (age, parity, marital status, educational level, residence, and household wealth index) were included.

Women parity, educational level, residence, and wealth index were significantly associated with late ANC booking. The odds of late ANC booking among grand multi-parous women was 1.35 times higher as compared to prim-parous women (AOR: 1.35, 95% CI (1.005, 1.83)). Likewise, the odds of late ANC booking among women who had attained primary school was decreased by 30% as compared to women who had no formal education (AOR: 0.70, 95%CI (0.58, 0.85)). Similarly, the odds of late ANC booking among women who had higher education was decreased by 75% as compared to women who had no formal education (AOR: 0.25, 95% CI (0.15, 0.40)). The odds of late ANC booking among women from rural resident was 1.62 times higher as compared to women from urban resident (AOR: 1.62, 95% CI (1.28, 2.05)). Antenatal booking was significantly associated with economic status of women’s households; the odds of late ANC booking among rich women was decreased by 31% as compared to poor women (AOR: 0.69, 95% CI (0.55, 0.85)) (**Table 4**).

**Table 4:** Multivariable logistic regression analysis of factors associated with late ANC booking among women in Ethiopia, 2019.

Variable	COR(95%CI)	AOR(95%CI)	P-value
**Parity**			
1(Rf)	1	1	
2–4	1.30(1.06–1.60)	1.16(0.91–1.48)	0.204
Five and more	2.06(1.66–2.67)	1.35(1.005–1.83)	0.046*
**Age (in years)**			
15–19 years(R_f_)	1	1	
20–29 years	0.75(0.53–1.04)	0.78(0.54–1.12)	0.187
30–39 years	0.90(0.64–1.27)	0.71(0.47–1.07)	0.108
40–49 years	1.10(0.72–1.68)	0.71(0.43, 1.18)	0.196
**Marital status**			
Never married(R_f_)	1	1	
Married	1.32(0.47–3.69)	0.87(0.30–2.56)	0.184
Widowed	1.68(0.48–5.84)	0.81(0.22–2.99)	0.758
Divorced	1.12(0.38–3.28)	0.84(0.27–2.58)	0.770
**Educational level**			
No education(R_f_)	1	1	
Primary	0.60(0.50–0.71)	0.70(0.58–0.85)	<0.001*
Secondary	0.33(0.25–0.45)	0.49(0.36–0.67)	<0.001*
Higher	0.14(0.09–0.22)	0.25(0.15–0.40)	<0.001*
**Residence**			
Urban(R_f_)	1	1	
Rural	2.63(2.17–3.18)	1.62(1.28–2.05)	<0.001*
**Wealth Index**			
Poor(R_f_)	1	1	
Middle	0.68(0.54–0.85)	0.74(0.58–0.93)	0.013*
Rich	0.74(0.66–0.84)	0.69(0.55–0.85)	0.001*

Notes:* p < 0.05; Rf = Reference category, Confidence intervals are in parenthesis

## Discussion

The current study was designed to investigate the utilization and quality of ANC services in Ethiopia. Furthermore, it aimed to identify factors associated with late ANC booking in Ethiopia using the nationally representative data. Regional disparities were observed on booking, utilization, and completion of ANC.

According to the survey, 74% of pregnant women received ANC services, with four out of ten not receiving the required (4+) ANC visits. This finding is higher than previous studies in Ethiopia (27.3%) [16], including 2016 EDHS report (62%, 32%) [4]. Furthermore, 68 percent of women booked ANC appointments early (before 4 months) during their previous pregnancy. This finding is higher than previous studies conducted in different parts of the country, ranging from 19–58% [17–23] and Zambia (28%) [24]. This difference could be because of the efforts made by health extension workers, health care providers and government commitment towards the improvement of maternal and child health services [25]. Moreover, the discrepancy might be due to the differences in study areas, period and small sample size of the previous studies.

In Ethiopia, the quality of ANC was poor; the fundamental components of ANC services that every pregnant woman should receive were not being provided as recommended. Only two-thirds of pregnant women who were on ANC follow-up were counseled about pregnancy danger signs of pregnancy, and 42% of pregnant women who were on ANC follow-up did not receive iron supplements.

In Ethiopia, ANC service utilization varied greatly by region, ranging from 25% in the Somali region to 96% in Addis Ababa city administration. Similarly, there were considerable differences in early ANC booking among the country’s regions and city administrations. This unacceptably low utilization as well as early booking in Somali and Afar regions might be due to the inequitable access to health services, lack of political commitment, and environmental hostility across regions [15].

This study also assessed factors associated with late ANC booking. It revealed that ANC booking was significantly associated with educational status of women. The odds of late booking was higher among women who have no formal education. This finding is supported by studies conducted in Ethiopia [15, 26, 27], Kenya [28] Nigeria [29], Ghana [30] and Colombia [31]. This is because education is a precursor for health-seeking behavior as educated women can access health-related information and have better decision-making power to get maternal health care services [15]. In this regard, education is vital to improve utilization of obstetric care in general and ANC in particular. Residence was significantly associated with late ANC booking; rural resident women book lately as compared to urban dwellers. Different studies in Ethiopia [27, 32, 33], Nigeria [34] and Myanmar [35] found out results consistent with ours. This is because women from rural area do not get appropriate maternal health care as a result of inadequate availability and accessibility of health services. Furthermore, ANC booking was also significantly associated with economic status of households; the likelihood of late ANC booking decreased as household economic status rose which is consistent with other studies in Ethiopia [32] and Nigeria [29]. Poverty negatively affects the ability of women to seek and access reproductive and maternal health services [15, 30, 36]. The study indicated that ANC booking was associated with parity; the probability of late booking was found to be higher among grand multi-parous women than prim-parous women. This finding is in agreement to a study conducted in Ethiopia [27, 32, 37] and Nairobi [28]. This could be because women who have many children are often preoccupied with caring for their children at home, leaving them with insufficient time to seek medical advice. In addition, the late initiation of ANC among grand multi-parous women may be due to past experiences. Finally, the study findings may be limited due to the absence of some healthcare service-related factors, such as distance to healthcare facilities that were not included in analyses due to their inaccessibility in the dataset.

## Conclusion and recommendation

Although utilization of ANC services in Ethiopia is improving, it is still far behind its targets, and a high proportion of pregnant women arrive late for ANC services. This study also highlighted the existence of a significant disparity in ANC services utilization among regions. Measures should be strengthened to improve utilization of quality ANC services, particularly for grand multi-parous women, and women from low economic status who live in rural areas and have low educational level. Equitable ANC services should be accessed by all regions of the country, with women from Somali and Afar regions receiving special attention.

## Supporting information

S1 Data Dataset used for the study of late antenatal care utilization in Ethiopia: The effect of socio-economic inequities and regional disparities. (DTA)

## Abbreviations

ANC: Antenatal Care
EA: Enumeration Area
EDHS: Ethiopian Demographic Health Survey
HSTP: Health Sector Transformation Plan
MMR: Maternal Mortality Ratio
OR: Odds Ratio
WHO: World Health Organization
